# Cyclization of Hydrazones of 2-Acetyl-1-naphthol and 1-Acetyl-2-naphthol with Triphosgene. Synthesis of Spiro Naphthoxazine Dimers

**DOI:** 10.3390/molecules14062147

**Published:** 2009-06-12

**Authors:** Abdullah Saad Al-Bogami, Abdullah Mohammed Al-Majid, Mohammed Ali Al-Saad, Ahmed Amine Mousa, Sara Abdullah Al-Mazroa, Hamad Zaid Alkhathlan

**Affiliations:** 1 Department of Chemistry, King Saud University, P. O. Box 2455, Riyadh 11451, Saudi Arabia; 2 Chemistry Department, Girls College of Education, Riyadh Girls University, Riyadh, Saudi Arabia

**Keywords:** 2-acetyl-1-naphthol, 1-acetyl-2-naphthol, triphosgene, naphthoxazine, spiro naphthoxazine

## Abstract

Cyclization of hydrazones derived from 2-acetyl-1-naphthol and 1-acetyl-2-naphthol with triphosgene gave naphtho[1,2-e]-1,3-oxazines, naphtho[2,1-e]-1,3-oxazines or their spiro dimers depending on the molar ratio of triphosgene used for the cyclization.

## Introduction

Triphosgene (bis(trichloromethyl)carbonate) has been repeatedly used in the literature for the construction of a variety of heterocyclic systems. It holds an advantage over other similar reagents, such as phosgene and diphosgene, of being a safe and easy to handle solid. Examples of important heterocyclic systems prepared using this reagent include benzothiadiazepines [1], quinazolines [2], diazolidines [3], imidiazolidines [4] and azetidines [5]. We have recently reported the use of triphosgene in the cyclization of hydrazones and Schiff bases of 2-hydroxy- and 2-aminoacetophenones to give 1,3-benzoxazines [6,7], spiro 1,3-benzoxazine dimmers [8], quinazolines and spiro quinazoline dimers [9].

We would like to report here results obtained from the cyclization of hydrazones of 2-acetyl-1-naphthol and 1-acetyl-2-naphthol with triphosgene.

## Results and Discussion

**Scheme 1:**
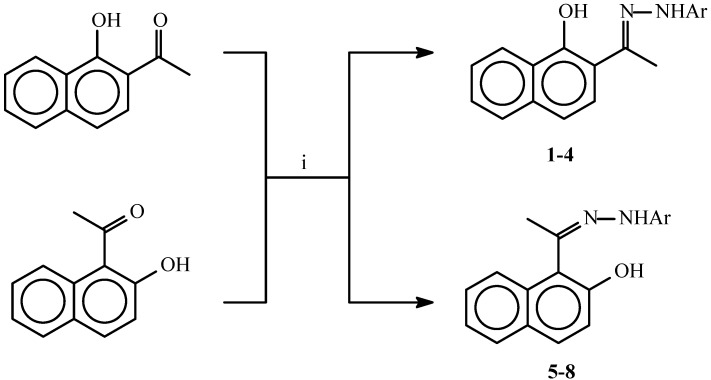
Synthesis of hydrazones of 2-acetyl-1-naphthol and 1-acetyl-2-naphthol.

Hydrazones of 2-acetyl-1-naphthol and 1-acetyl-2-naphthol were obtained in very good yields from the reaction of these compounds with aromatic hydrazines (Scheme 1, Table 1).

i = ArNHNH_2_.HCl/EtOH/AcONa/H_2_O

**Table 1:** Hydrazones **1-8**.

Compound	Ar	Yield (%)
**1**	Phenyl	92
**2**	4-ClC_6_H_4_	82
**3**	4-BrC_6_H_4_	79
**4**	4-CH_3_C_6_H_4_	91
**5**	Phenyl	75
**6**	4-ClC_6_H_4_	80
**7**	4-BrC_6_H_4_	68
**8**	4-CH_3_C_6_H_4_	68

**Scheme 2:**
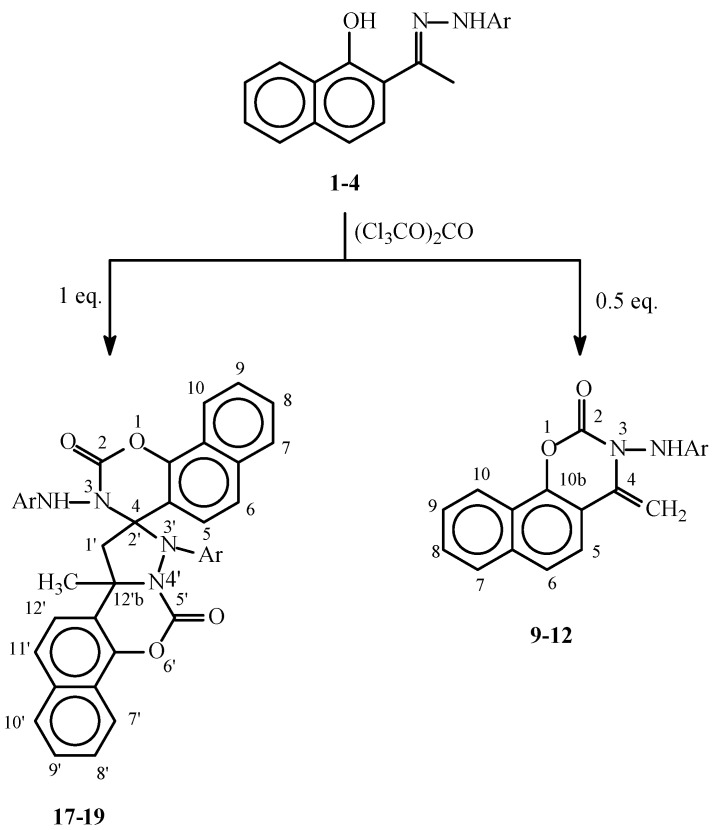
Reaction of hydrazones 1-4 with triphosgene.

**Scheme 3:**
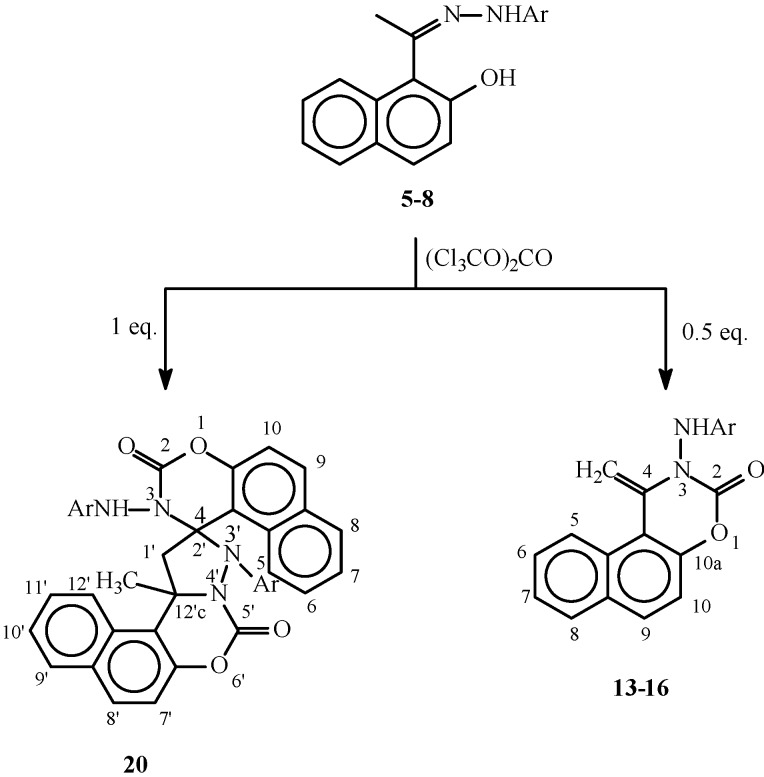
Reaction of hydrazones 5-8 with triphosgene.

The hydrazones **1-8** show in the their IR spectra absorbances for the C=N group in the 1,614-1,636 cm^-1^ range and for the OH and NH groups at about 3,400 cm^-1^ and 3,350 cm^-1^, respectively. The ^1^H-NMR spectra of compounds **1-8** show in each case two distinct doublets which have been assigned to the H_3_ and H_4_ positions. In the spectra of **1-4** these two doublets appear at δ 7.04-7.16 and δ 7.68-7.76. The lower field signal in this case was assigned to H_3_ as it is deshielded by the hydrazone moiety. In contrast, for compounds **5-8**, where the two doublets appear at δ 6.80-6.90 and δ 7.63-7.66 the signal at the higher field was assigned to H_3_ as in this case this position is shielded by the OH group. It is also worth mentioning here that in the ^13^C-NMR spectra of the above compounds, the methyl group of the hydrazones of the 2-acetyl derivatives **1-4** absorbs at higher fields (δ 12.22-13.04) than those of the 1-acetyl derivatives **5-8** (δ 18.29-18.55). The rest of the spectral data is shown in the Experimental section. Treatment of the hydrazones **1-8** with triphosgene gave the 4-methylenenaphthoxazines **9-16** or the spiro naphthoxazine dimers **17-20**, depending on the molar ratio of triphosgene used in the cyclizations (Scheme 2 and Scheme 3, Table 2).

**Table 2:** 4-Methylenenaphthoxazines **9-16** and spiro naphthoxazine dimmers **17-20**.

Compound	Ar	Yield (%)
**9**	Phenyl	69
**10**	4-ClC_6_H_4_	74
**11**	4-BrC_6_H_4_	71
**12**	4-CH_3_C_6_H_4_	73
**13**	Phenyl	62
**14**	4-ClC_6_H_4_	61
**15**	4-BrC_6_H_4_	64
**16**	4-CH_3_C_6_H_4_	60
**17**	Phenyl	69
**18**	4-ClC_6_H_4_	72
**19**	4-BrC_6_H_4_	71
**20**	4-CH_3_C_6_H_4_	55

These results are in agreement with our previous reports on the reaction of hydrazones of 2-hydroxyacetophenones with triphosgene. It was observed then that the use of 0.5 equivalents of triphosgene gave the 4-methylene-1,3-benzoxazines, while the spiro 1,3-benzoxazine dimers were obtained when 1.0 equivalent of triphosgene was used [8]. The structures of those compounds were established based on X-ray crystal structure and spectroscopic studies [8,9]. As a result, we are showing here partial spectroscopic data for compounds **9-16** (Table 3 and Table 4) and **17-20** (Table 5). In these tables, signals that can be easily attributed to structurally related positions, and with significance to the elucidation of the structures of the new compounds are shown for matter of comparison, while the complete data is given in the experimental section.

**Table 3:** Partial IR and NMR data for compounds **9-16**.

Compound	IR (cm^-1^)		^1^H-NMR (ppm)	
	C=O	=CH_2_	=CH_2_	Aromatic
**9**	1,727	1,620	5.02 (d, *J* = 2.2 Hz, 1H)	6.80 (d, *J* = 8.1 Hz, 2H, H_2,6,Ar_)
			5.13 (d, *J* = 2.2 Hz, 1H)	7.61 (d, *J* = 8.8 Hz, 1H, H_6_)
				7.66 (d, *J* = 8.8 Hz, 1H, H_5_)
**10**	1,732	1,621	5.02 (d, *J* = 2.2 Hz,1H)	6.78 (d, *J* = 9.0 Hz, 2H, H_2,6,Ar_)
			5.09 (d, *J* = 2.2 Hz, 1H)	7.23 (d, *J* = 9.0 Hz,2H, H_3,5,Ar_)
				7.67 (d, *J* = 8.8 Hz, 1H, H_5_)
**11**	1,741	1,621	4.96 (d, *J* = 2.0 Hz, 1H)	6.73 (d, *J* = 8.8 Hz, 2H, H_2,6,Ar)_
			5.01 (d, *J* = 2.0 Hz, 1H)	7.24 (d, *J* = 8.8 Hz, 2H, H_3,5,Ar_)
**12**	1,737	1,612	4.92 (d, *J* = 2.0 Hz, 1H)	6.71 (d, *J* = 8.1 Hz, 2H, H_2,6,Ar_)
			5.14 (d, *J* = 2.0 Hz, 1H)	7.02 (d, *J* = 8.1 Hz, 2H, H_3,5,Ar_)
				7.84 (d, *J* = 8.8 Hz, 1H, H_6_)
				7.91 (d, *J* = 8.8 Hz, 1H, H_5_)
**13**	1,725	1,620	5.04 (d, *J* = 2.2 Hz, 1H)	6.97 (d, *J* = 8.8 Hz, 2H, H_2,6,Ar_)
			5.15 (d, *J* = 2.2Hz, 1H)	7.67 (d, *J* = 8.1 Hz, 1H, H_9_)
**14**	1,732	1,620	5.05 (d, *J* = 1.5Hz, 1H)	6.84 (d, *J* = 8.8 Hz, 2H, H_2,6,Ar_)
			5.15 (d, *J* = 1.5 Hz, 1H)	7.23 (d, *J* = 8.8 Hz, 2H, H_3,5,Ar_)
				7.70 (d, *J* = 8.1 Hz, 1H, H_9_)
**15**	1,733	1,616	5.05 (d, *J* = 2.2 Hz, 1H)	6.90 (d, *J* = 8.0 Hz, 2H, H_2,6,Ar_)
			5.14 (d, *J* = 2.2 Hz, 1H)	7.29 (d, *J* = 8.0 Hz, 2H, H_3,5,Ar_)
**16**	1,741	1,615	5.04 (d, *J* = 2.2 Hz, 1H)	6.81(d, *J* = 8.8 Hz, 2H, H_2,6,Ar_)
			5.14 (d, *J* = 2.2 Hz, 1H)	7.08 (d, *J* = 8.8 Hz, 2H, H_3,5,Ar_)
				7.66 (d, *J* = 8.8 Hz, 1H, H_9_)

**Table 4:** Partial ^13^C-NMR data for **9-16**.

	9	10	11	12	13	14	15	16
C_1,Ar_	143.05	143.46	143.35	143.34	143.53	143.48	143.53	142.88
C_2,6,Ar_	113.03	115.70	114.89	113.07	113.97	115.33	113.96	114.12
C_3,5,Ar_	129.30	132.33	131.99	130.03	129.45	129.44	129.47	129.96
C_2_	148.05	147.99	147.35	147.21	148.07	148.01	148.08	148.10
C_4_	138.69	138.26	138.54	138.99	138.46	138.30	138.45	138.48
=CH_2_	88.60	88.55	88.58	88.97	88.53	88.53	88.54	88.50
C_5_ (C_10_)	122.93	121.48	121.52	121.50	(120.33)	(120.25)	(120.33)	(120.34)
C_6_ (C_9_)	120.69	120.24	120.73	121.05	(128.12)	(128.23)	(128.13)	(128.10)
C_10b_ (C_10a_)	146.16	144.45	145.60	144.14	(145.29)	(143.95)	(145.29)	(143.53)
C_2_	148.05	147.99	147.35	147.21	148.07	148.01	148.08	148.10

**Table 5:** Partial ^1^H and ^13^C-NMR data for the spiro compounds **17- 20**.

	^1^H		^13^C				
	CH_3_	C_1`_	CH_3_	C_1`_	C_2`_	C_12`b_	C=O
**17**	2.12	3.58 (d, *J* = 14.70 Hz, 1H)	32.15	59.65	85.55	66.06	149.24
		3.69 (d, *J* = 14.70 Hz, 1H)					149.90
**18**	2.10	3.49 (d, *J* = 14.70 Hz, 1H)	32.08	60.07	85.47	66.05	149.01
		3.60 (d, *J* = 14.70 Hz, 1H)					149.07
**19**	2.09	3.44 (d, *J* = 14.70 Hz, 1H)	32.04	60.11	85.41	65.96	148.95
		3.54 (d, *J* = 14.70 Hz, 1H)					149.15
**20**	2.51	3.41 (d, *J* = 14.65 Hz, 1H)	17.20	27.87	97.68	(88.06)*	148.60
		3.54 (d, *J* = 14.65 Hz, 1H)					149.01

*(C_12'c_).

It can be seen from Table 3 that there is a noticeable difference in the ^13^C-NMR spectra of **17-19** compared with **20**. In the latter compound the CH_3_ group and position C_1`_ absorb at higher fields than those in **17-19**, while positions C_2`_ and C_12`c_ absorb at lower fields than positions C_2'_ and C_12'b_ in **17-19**. This might be speculated as a result of the orientation of the aromatic rings in respect to these positions, which needs further studies. In addition, the above results for compounds **17-19** are in close resemblance to these reported for the spiro 1,3-benzoxazine dimmers which might suggest that they have similar steric environment for the 1,3-benzoxazines [8].

The EIMS spectra of the spiro compounds **17-20** did not show the expected molecular ions. They appear to fragment at the spiro junction to give fragments at m/z = M^+^ - ArN_2_CO and m/z = M^+^ ‑ ArN_2_CO_2_. For example, compound **18** shows in its EIMS spectra fragment peaks at m/z 505 and 489, arising from the loss of ClC_6_H_4_N_2_CO and ClC_6_H_4_N_2_CO_2_, respectively. This type of fragmentation was previously observed with analogous spiro compounds [8,9].

Finally, it is worth mentioning here that although the formation of the 4-methylene-naphthoxazines **9-16** would be expected to proceed via a similar reaction mechanism to that previously reported for the cyclization of hydrazones of 2-hydroxyacetophenone and acetophenone [6,10], the mechanism for formation of the spiro naphthoxazine dimmers is not clear to us with regards to at what stage does the dimerization occurs to form the final spiro naphthoxazines.

**Scheme 4:**
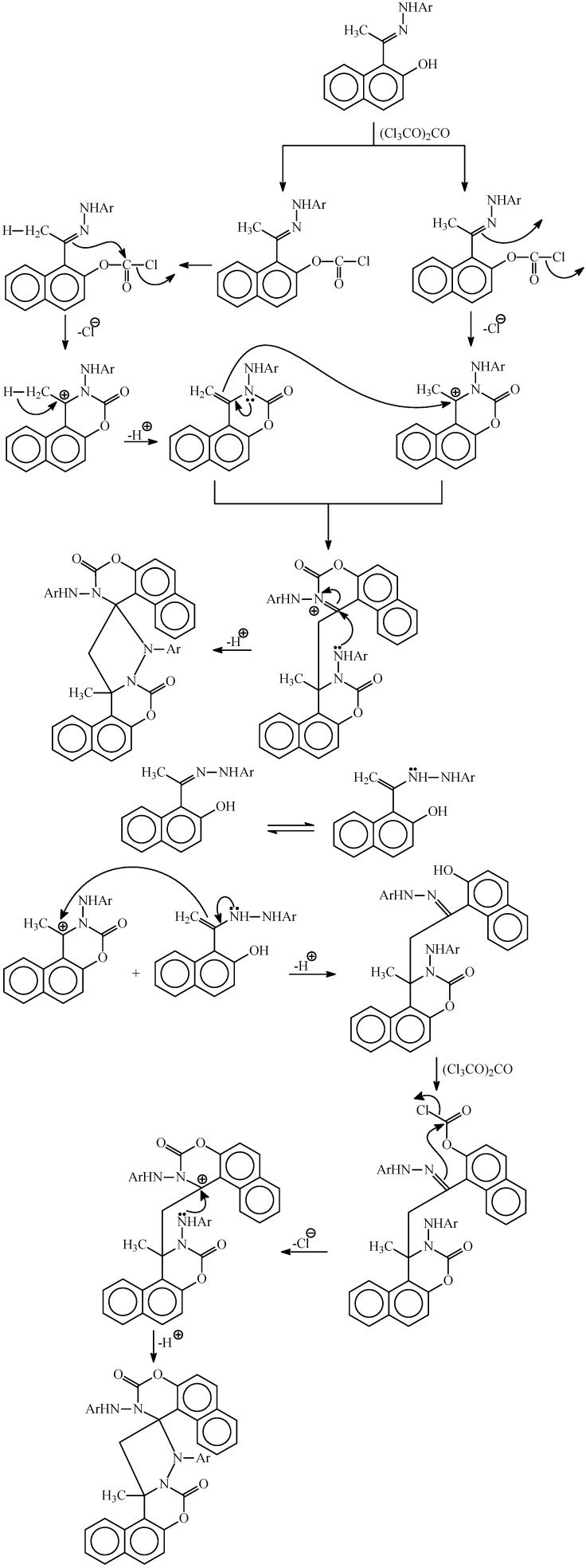
Proposed mechanism for the formation of the spiro compounds **17-20**.

The use of 1.0 equivalents of triphosgene is necessary to form the latter products because in its reactions it gives an equivalent of three moles of phosgene (COCl_2_), so the formation of naphthoxazines (where one CO group is introduced) would require only 0.5 equivalent of triphosgene (1.5 equivalent of COCl_2_, a slight excess is usually used in these kinds of cyclizations) while the spiro dimers (where two CO groups are introduced) would require double this amount. In Scheme 4, a speculative mechanism for the formation of the spiro products is shown with two pathways; in the first one the dimerization occurs after the formation of the two naphthoxazine nuclei, while in the second one the dimerization takes place before the formation of the second naphthoxazine ring.

## Experimental

Melting points are uncorrected. IR spectra were recorded on a Perkin-Elmer 883 spectrophotometer as KBr pellets and expressed as ν in cm^-1^. NMR spectra were recorded on JEOL ECP 400 (400 MHz) in CDCl_3_ and chemical shifts are expressed as δ in ppm. MS spectra were recorded on Shimadzu QP 5050A GC/MS system.

*2-Acetyl-1-naphthol phenylhydrazone* (**1**): To a solution of 2-acetyl-1-naphthol (2.0 g, 10.8 mmol) in ethanol (50 mL) was added a solution of phenylhydrazine hydrochloride (1.9 g, 12.9 mmol) and sodium acetate (1.0 g, 12.2 mmol) in a mixture of ethanol (30 mL) and water (10 mL). The above solution was refluxed for 5 hours and then evaporated under vacuum. The resulting solid was washed with water (50 mL) and then recrystallized from ethanol-chloroform (8:2). Yield 92%, red solid, mp 162°C; IR: 3,410 (OH), 3,334 (NH), 1,620 (C=N); ^1^H-NMR: 1.81 (s, 3H), 6.17 (t, *J* = 7.3 Hz, 1H), 6.46 (d, *J* = 7.7 Hz, 2H), 6.62 (m, 3H), 6.77 (m, 2H), 6.88 (d, *J* = 8.8 Hz, 1H), 7.06 (m, 1H), 7.64 (m, 1H); ^13^C-NMR: 13.04, 112.42, 114.49, 117.42, 119.49, 122.49, 123.58, 124.66, 126.51, 126.75, 128.60, 128.84, 133.41, 144.69, 148.03, 154.09; MS: *m/z* (%) 276 (M^+^, 100), 261 (7), 260 (18), 295 (38), 230 (6), 183 (25), 168 (6), 141 (13), 128 (10), 115 (49), 77 (8); Anal. Calcd. for C_18_H_16_N_2_O: C, 78.23; H, 5.83; N, 10.13. Found: C, 77.96; H, 5.71; N, 10.28.

*2-Acetyl-1-naphthol 4̀-chlorophenylhydrazone* (**2**): This compound was prepared from 2-acetyl-1-naphthol and 4̀-chlorophenylhydrazine using the procedure described for **1**. Yield, 82%, brownish solid, mp 175°C; IR: 3,420 (OH), 3,331 (NH), 1,636 (C=N); ^1^H-NMR: 2.38 (s, 3H), 6.98 (d, *J* = 8.8 Hz, 2H), 7.26 (d, *J* = 8.8 Hz, 2H), 7.32 (m, 2H), 7.49 (m, 2H), 7.73 (m, 1H), 8.40 (m, 1H); ^13^C-NMR: 12.28, 112.67, 114.27, 118.24, 123.22, 123.51, 125.37, 125.67, 127.15, 127.34, 129.49, 134.28, 142.68, 149.27, 155.67; MS: m/z (%) 312 (M+2, 21), 310 (M^+^, 65), 296 (5), 294 (10), 293 (51), 258 (17), 184 (30), 183 (41), 169 (10), 168 (9), 143 (22), 141 (25), 134 (45), 127 (100), 114 (37), 76 (6); Anal. Calcd. for C_18_H_15_ClN_2_O: C, 69.56; H, 4.86; N, 9.01. Found: C, 69.72; H, 4.77; 8.88.

*2-Acetyl-1-naphthol 4̀-bromophenylhydrazone* (**3**): This compound was prepared from 2-acetyl-1-naphthol and 4̀-bromophenylhydrazine using the procedure described for **1**. Yield, 79%, brownish solid, mp 182°C; IR: 3,412 (OH), 3,348 (NH), 1,632 (C=N); ^1^H-NMR: 2.40 (s, 3H), 6.94 (d, *J* = 8.1 Hz, 2H), 7.31 (d, *J* = 8.8 Hz, 1H), 7.41 (d, *J* = 8.8 Hz, 1H), 7.48 (d, *J* = 8.1 Hz, 2H), 7.53 (m, 2H), 7.74 (m, 1H), 8.41 (m, 1H); ^13^C-NMR: 12.29, 112.92, 113.20, 118.20, 121.12, 123.32, 123.63, 125.33, 125.38, 127.23, 127.30, 129.70, 134.27, 144.11, 148.52, 155.12; MS: m/z (%) 356 (M+2, 70), 354 (M^+^, 71), 340 (11), 339 (54), 338 (12), 337 (49), 258 (21), 185 (15), 183 (56), 173 (63), 156 (10), 128 (18), 115 (100), 91 (35), 76 (6); Anal. Calcd. for C_18_H_15_BrN_2_O: C, 60.86; H, 4.25; N, 7.88. Found: C, 60.67; H, 4.15; N, 7.75.

*2-Acetyl-1-naphthol 4̀-methylphenylhydrazone* (**4**): This compound was prepared from 2-acetyl-1-naphthol and 4̀-methylphenylhydrazene using the procedure described for **1**. Yield, 91% brownish solid, mp 164°C; IR: 3,424 (OH), 3,350 (NH), 1,614 (C=N); ^1^H-NMR: 2.01 (s, 3H), 2.19 (s, 3H), 6.76 (d, *J* = 8.1 Hz, 2H), 6.80 (d, *J* = 8.1 Hz, 2H), 7.02 (m, 1H), 7.17 (m, 2H), 7.24 (m, 1H), 7.45 (m, 1H), 8.06 (m, 1H); ^13^C-NMR: 12.22, 20.73, 113.03, 113.31, 118.13, 118.42, 123.31, 123.62, 125.01, 125.32, 127.22, 127.49, 130.15, 134.21, 141.96, 148.05, 155.06; MS: m/z (%) 290 (M^+^, 85), 275 (14), 273 (54), 185 (15), 168 (12), 128 (6), 115 (37), 107 (100), 93 (6), 76 (25); Anal. Calcd. for C_19_H_18_N_2_O: C, 78.59; H, 7.95; N, 12.27. Found: C, 78.38; H, 7.85; N, 12.12.

*1-Acetyl-2-naphthol phenylhydrazone* (**5**): This compound was prepared from 1-acetyl-2-naphthol and phenylhydrazine hydrochloride using the procedure described for **1**. Yield, 75%, pale yellow solid, mp 125°C; IR: 3,418 (OH), 3,335 (NH), 1,617 (C=N); ^1^H-NMR: 2.49 (s, 3H), 6.93 (t, *J* = 7.3Hz, 1H), 7.12 (d, *J* = 7.3 Hz, 2H), 7.21 (d, *J* = 8.8 Hz, 1H), 7.29 (d, *J* = 7.3 Hz, 2H), 7.33 (m, 1H), 7.44 (m, 1H), 7.79 (d, *J* = 8.1 Hz, 1H); ^13^C-NMR: 18.41, 113.18, 116.32, 118.64, 121.13, 123.15, 124.52, 126.50, 128.97, 129.26, 129.63, 130.77, 131.66, 144.24, 144.76, 153.43; MS: m/z (%) 276 (M^+^, 69), 260 (21), 259 (100), 184 (22), 169 (6), 141 (9), 128 (7), 115 (28), 93 (33), 77 (9); Anal. Calcd. for C_18_H_16_N_2_O: C, 78.23, H, 5.73, N, 10.13. Found: C, 78.11; H, 5.73, N, 10.08.

*1-Acetyl-2-naphthol 4̀-chlorophenylhydrazone* (**6**): This compound was prepared from 1-acetyl-2-naphthol and 4̀-chlorophenylhydrazine using the procedure described for **1**. Yield, 80%, pale yellow solid, mp 154°C; IR: 3,422 (OH), 3,355 (NH), 1,619 (C=N); ^1^H-NMR: 2.47 (s, 3H), 7.04 (d, *J* = 9.2 Hz, 2H), 7.21 (d, *J* = 9.2 Hz, 2H), 7.24 (d, *J* = 8.8, 1H), 7.33 (m, 1H), 7.44 (m, 1H), 7.66 (d, *J* = 8.4 Hz, 1H), 7.72 (d, *J* = 8.8 Hz, 1H), 7.77 (d, *J* = 7.7 Hz, 1H); ^13^C-NMR: 18.42, 114.36, 116.29, 118.57, 123.24, 124.44, 126.60, 129.00, 129.05, 129.30, 129.51, 130.94, 131.65, 142.93, 143.11, 153.30; MS: m/z (%) 312 (M+2, 21), 310 (M^+^, 62), 296 (7), 295 (34), 294 (23), 293 (100), 184 (45), 183 (18), 169 (16), 168 (9), 141 (24), 127 (74), 76 (6); Anal. Calcd. for C_18_H_15_ClN_2_O: C, 69.56; H, 4.86; N, 9.01. Found: C, 69.42; H, 4.73; N, 9.12.

*1-Acetyl-2-naphthol 4̀-bromophenylhydrazone* (**7**): This compound was prepared from 1-acetyl-2-naphthol and 4̀-bromophenylhydrazine using the procedure described for **1**. Yield, 68%, yellow solid, mp 153°C; IR: 3,436 (OH), 3,354 (NH), 1,619 (C=N); ^1^H-NMR: 2.49 (s, 3H), 7.01 (d, *J* = 8.8 Hz, 2H), 7.24 (d, *J* = 8.4 Hz, 1H), 7.34 (m, 1H), 7.39 (d, *J* = 8.8 Hz, 2H), 7.45 (m, 1H), 7.67 (d, *J* = 8.4 Hz, 1H), 7.73 (d, *J* = 8.8 Hz, 1H), 7.78(d, *J* = 8.4 Hz, 1H); ^13^C-NMR: 18.29, 112.86, 114.66, 116.23, 118.48, 123.14, 124.35, 126.49, 128.90, 129.20, 130.82, 131.54, 132.31, 143.29, 145.04, 153.19; MS: m/z (%) 356 (M+2, 71), 354 (M^+^, 75), 340 (19), 339 (97), 338 (22), 337 (100), 258 (8), 210 (17), 185 (12), 183 (27), 173 (44), 169 (67), 115 (87), 76 (7); Anal. Calcd. for C_18_H_15_BrN_2_O: C, 60.86; H, 4.25; N, 7.88. Found: C, 60.73; H, 4.38; N, 7.93.

*1-Acetyl-2-naphthol 4̀-methylphenylhydrazone* (**8**): This compound was prepared from 1-acetyl-2-naphthol and 4̀-methylphenylhydrazine using the procedure described for **1**. Yield, 68%, yellow solid, mp 132 °C; IR: 3,417 (OH), 3,360 (NH), 1,618 (C=N); ^1^H-NMR: 2.29 (s, 3H), 2.49 (s, 3H), 7.02 (d, *J* = 8.1 Hz, 2H), 7.09 (d, *J* = 8.1 Hz, 2H), 7.21 (d, *J* = 8.8 Hz, 1H), 7.32 (m, 1H), 7.44 (m, 1H), 7.68 (d, *J* = 8.1 Hz, 1H), 7.72 (d, *J* = 8.8 Hz, 1H), 7.78 (d, *J* = 7.3 Hz, 1H); ^13^C-NMR: 18.55, 20.69, 113.38, 116.22, 118.66, 123.15, 124.51, 126.51, 128.96, 129.24, 130.09, 130.62, 130.81, 131.65, 142.05, 143.11, 153.54; MS: m/z (%) 290 (M^+^, 89), 273 (100), 184 (30), 169 (7), 141 (11), 128 (8), 115 (35), 107 (57), 106 (36), 105 (46), 91 (6), 76 (25); Anal. Calcd. for C_19_H_18_N_2_O: C, 78.59; H, 7.95; N, 12.27. Found: C, 78.41; H, 7.81; N, 12.18.

*4-Methylene-3-(N-phenylamino)-3,4-dihydro-2H-naphth[2,1-e]-1,3-oxazine-2-one* (**9**): To a stirred solution of 0.20 g (0.72 mmol) of **1** and triethylamine (1 mL) in dichloromethane (30 mL) was added dropwise under N_2_ atmosphere a solution of 0.5 equivalent (0.12 g, 0.36 mmol) of triphosgene in dichloromethane (10 mL). The mixture was refluxed for 4 hours and then washed with water. The organic layer was dried over magnesium sulfate and evaporated under vacuum. The resulting solid was recrystallized from a mixture of benzene-ethanol (1:1). Yield, 69%, colorless solid, mp 196°C; IR: 3,306 (NH), 1,727 (C=O), 1,620 (=CH_2_); ^1^H-NMR: 5.02 (d, *J* = 2.2 Hz, 1H), 5.13 (d, *J* = 2.2 Hz, 1H), 6.80 (d, *J* = 8.1 Hz, 2H), 7.02 (m, 3H), 7.55 (m, 2H), 7.61 (d, *J* = 8.8 Hz, 1H), 7.66 (d, *J* = 8.8 Hz, 1H), 7.83 (m, 1H), 8.20 (m, 1H), 8.33 (bs, 1H); ^13^C-NMR: 88.60, 111.50, 113.03, 120.69, 121.57, 122.93, 125.18, 127.46, 127.89, 128.12, 128.60, 129.30, 134.57, 138.69, 143.05, 146.16, 148.05; MS: m/z (%) 302 (M^+^, 13), 260 (25), 259 (100), 232 (11), 230 (23), 182 (18), 168 (11), 140 (9), 139 (17), 128 (25), 92 (13), 77 (9); Anal. Calcd. for C_19_H_14_N_2_O_2_: C, 75.48; H, 4.67; N, 9.27. Found: C, 75.13; H, 4.50; N, 9.63.

*4-Methylene-3-[N-(4̀-chlorophenyl)amino]-3,4-dihydro-2H-naphth[2,1-e]-1,3-oxazine-2-one* (**10**): Prepared from **2** and triphosgene using the procedure described for **9**. Yield, 74%, pale yellow solid, mp 182°C; IR: 3,260 (NH), 1,732 (C=O), 1,621 (=CH_2_); ^1^H-NMR: 5.02 (d, *J* = 2.2 Hz, 1H), 5.09 (d, *J* = 2.2 Hz, 1H), 6.57 (bs, 1H), 6.78 (d, *J* = 9.0 Hz, 2H), 7.23 (d, *J* = 9.0 Hz, 2H), 7.59 (m, 3H), 7.67 (d, *J* = 8.8 Hz, 1H), 7.85 (m, 1H), 8.30 (m, 1H); ^13^C-NMR: 88.55, 111.09, 114.46, 115.70, 120.24, 121.48, 123.03, 125.36, 127.53, 127.70, 128.22, 132.33, 134.68, 138.26, 143.46, 144.45, 147.99; MS: m/z (%) 338 (M+2, 6), 336 (M^+^, 17), 301 (24), 296 (9), 295 (32), 294 (23), 293 (91), 259 (28), 258 (100), 230 (17), 182 (53), 140 (18), 128 (74), 115 (21), 98 (31), 76 (10); Anal. Calcd. for C_19_H_13_Cl N_2_O_2_: C, 67.76; H, 3.89; N, 8.31. Found: C, 67.47; H, 3.71; N, 8.19.

*4-Methylene-3-[N-(4̀-bromophenyl)amino]-3,4-dihydro-2H-naphth[2,1-e]1,3-oxazine-2-one* (**11**): Obtained from **3** and triphosgene using the procedure described for **9**. Yield, 71%, brownish solid, mp 185°C; IR: 3,281 (NH), 1,741 (C=O), 1,621 (=CH_2_); ^1^H-NMR: 4.96 (d, *J* = 2.0 Hz, 1H), 5.01 (d, *J* = 2.0 Hz, 1H), 6.73 (d, *J* = 8.8 Hz, 2H), 7.24 (d, *J* = 8.8 Hz, 2H), 7.54 (m, 4H), 7.83 (m, 1H), 8.17 (m, 1H), 8.69 (bs, 1H); ^13^C-NMR: 88.58, 111.33, 112.06, 114.89, 120.73, 121.52, 122.88, 125.27, 127.53, 127.95, 128.21, 131.99, 134.59, 138.54, 143.35, 145.60, 147.35; MS: m/z (%) 382 (M+2, 12), 380 (M^+^, 13), 354 (12), 340 (16), 339 (74), 337 (72), 301 (44), 259 (36), 258 (100), 230 (26), 182 (58), 128 (74), 115 (33), 91 (43), 76 (11); Anal. Calcd. for C_19_H_13_BrN_2_O_2_: C, 59.86; H, 3.43; N, 7.34. Found: C, 59.73; H, 3.33; N, 7.43.

*4-Methylene-3-[N-(4-methylphenyl)amino]-3,4-dihydro-2H-naphth[2,1-e]-1,3-oxazine-2-one* (**12**): Obtained from **4** and triphosgene using the procedure described for **9**. Yield, 73%, brownish solid, mp 150°C; IR: 3,260 (NH), 1,737 (C=O), 1,612 (=CH_2_); ^1^H-NMR: 2.19 (s, 3H), 4.92 (d, *J* = 2.0 Hz, 1H), 5.14 (d, *J* = 2.0 Hz, 1H), 6.71 (d, *J* = 8.1 Hz, 2H), 7.02 (d, *J* = 8.1 Hz, 2H), 7.69 (m, 2H), 7.84 (d, *J* = 8.8 Hz, 1H), 7.91 (d, *J* = 8.8 Hz, 1H), 8.04 (m, 1H), 8.17 (m, 1H), 8.67 (bs, 1H); ^13^C-NMR: 20.72, 88.97, 111.70, 113.07, 121.50, 122.80, 125.43, 128.10, 128.43, 128.62, 129.14, 130.03, 134.66, 138.99, 143.34, 144.14, 147.21; MS: m/z (%) 316 (M^+^, 24), 274 (20), 273 (100), 244 (10), 182 (13), 128 (23), 105 (61), 76 (31); Anal. Calcd. for C_20_H_16_N_2_O_2_: C, 75.93; H, 5.10; N, 8.85. Found: C, 75.81; H, 5.15; N, 8.77.

*4-Methylene-3-(N-phenylamino)-3,4-dihydro-2H-naphth[1,2-e]-1,3-oxazine-2-one* (**13**): Obtained from **5** and triphosgene using the procedure described for **9**. Yield, 62%, colorless solid, mp 166 °C; IR: 3,297 (NH), 1,725 (C=O), 1,620 (=CH_2_); ^1^H-NMR: 5.04 (d, *J* = 2.2 Hz, 1H), 5.15 (d, *J* = 2.2 Hz, 1H), 6.53 (bs, 1H), 6.97 (d, *J* = 8.8 Hz, 2H), 7.25 (m, 3H), 7.58 (m, 3H), 7.67 (d, *J* = 8.1 Hz, 1H), 7.84 (m, 1H), 8.32 (m, 1H); ^13^C-NMR: 88.53, 111.24, 113.97, 120.33, 121.90, 122.27, 123.10, 125.23, 127.44, 127.67, 128.12, 129.45, 134.66, 138.46, 143.53, 145.29, 148.07; MS: m/z (%) 302 (M^+^, 7), 258 (100), 182 (16), 128 (5), 115 (7), 92 (5), 77 (6); Anal. Calcd. for C_19_H_14_N_2_O_2_ : C, 75.48; H, 4.67; N, 9.27. Found: C, 75.24; H, 4.71; N, 9.18.

*4-Methylene-3-[N-(4-chlorophenyl)amino]-3,4-dihydro-2H-naphth[1,2-e]-1,3-oxazine-2-one* (**14**): **O**btained from **6** and triphosgene using the procedure described for **9**. Yield, 61%, brownish solid, mp 187°C; IR: 3,260 (NH), 1,732 (C=O), 1,620 (=CH_2_); ^1^H-NMR: 5.05 (d, *J* = 1.5 Hz, 1H), 5.15 (d, *J* = 1.5 Hz, 1H), 6.52 (bs, 1H), 6.84 (d, *J* = 8.8 Hz, 2H), 7.23 (d, *J* = 8.8 Hz, 2H), 7.51 (m, 3H), 7.70 (d, *J* = 8.1 Hz, 1H), 7.86 (m, 1H), 8.31 (m, 1H); ^13^C-NMR: 88.53, 111.10, 115.33, 120.25, 121.87, 123.04, 125.37, 127.22, 127.54, 127.71, 128.23, 129.44, 134.69, 138.30, 143.48, 143.95, 148.01; MS: m/z (%) 338 (M+2, 5), 336 (M^+^, 14), 301 (24), 295 (29), 259 (29), 258 (100), 230 (14), 182 (60), 128 (68), 115 (21), 99 (36), 76 (9); Anal. Calcd. for C_19_H_13_Cl N_2_O_2_: C, 67.76; H, 3.89; N, 8.32. Found: C, 67.59; H, 3.95; N, 8.25.

*4-Methylene-3-[N-(4-bromophenyl)amino]-3,4-dihydro-2H-naphth[1,2-e]-1,3-oxazine-2-one* (**15**): Obtained from **7** and triphosgene using the procedure described for **9**. Yield, 64%, colorless solid, mp 203°C; IR: 3,353 (NH), 1,733 (C=O), 1,616 (=CH_2_); ^1^H-NMR: 5.05 (d, *J* = 2.2 Hz, 1H), 5.14 (d, *J* = 2.2 Hz, 1H), 6.53 (bs, 1H), 6.90 (d, *J* = 8.0 Hz, 2H), 7.29 (d, *J* = 8.0 Hz, 2H), 7.60 (m, 3H), 7.72 (d, *J* = 8.8 Hz, 1H), 7.82 (m, 1H), 8.12 (m, 1H); ^13^C-NMR: 88.54, 111.24, 113.96, 116.78, 120.33, 121.90, 125.24, 127.16, 127.45, 127.68, 128.13, 129.47, 134.66, 138.45, 143.53, 145.29, 148.08; MS: m/z (%) 382 (M+2, 18), 380 (M^+^, 16), 301 (35), 259 (30), 258 (100), 230 (35), 210 (18), 184 (49), 182 (48), 139 (45), 126 (69), 115 (72), 101 (35), 76 (19); Anal. Calcd. for C_19_H_13_BrN_2_O_2_: C, 59.86; H, 3.44; N, 7.35. Found: C, 59.98; H, 3.18; N, 7.12.

*4-Methylene-3-[N-(4-methylphenyl)amino]-3,4-dihydro-2H-naphth[1,2-e]-1,3-oxazine-2-one* (**16**): Obtained from **8** and triphosgene using the procedure described for **9**. Yield 60%, yellowish solid, mp 160°C; IR: 3,245 (NH), 1,741 (C=O), 1,615 (=CH_2­_); ^1^H-NMR: 2.26 (s, 3H), 5.04 (d, *J* = 2.20 Hz, 1H), 5.14 (d, *J* = 2.20 Hz, 1H), 6.47 (bs, 1H), 6.81 (d, *J* = 8.8 Hz, 2H), 7.08 (d, *J* = 8.8 Hz, 2H), 7.58 (m, 3H), 7.66 (d, *J* = 8.8 Hz, 1H), 7.85 (m, 1H), 8.32 (m, 1H); ^13^C-NMR: 20.71, 88.50, 111.28, 114.12, 120.34, 121.91, 123.10, 125.19, 127.43, 127.67, 128.10, 129.96, 131.73, 134.63, 138.48, 142.88, 143.53, 148.10; MS: m/z (%) 316 (M^+^, 13) 274 (17), 273 (100), 182 (11), 139 (15), 128 (20), 106 (52), 76 (36); Anal. Calcd. for C_20_H_16_N_2_O_2_: C, 75.93; H, 5.10; N, 8.85. Found: C, 75.75; H, 5.22; N, 8.73.

*3’-Phenyl-3-(phenylamino)-1’,12’b-dihydro-12`b-methylspiro{4H-naphth[2,1-e]-1,3-oxazine-4,2’ (3’H)- [5H]pyrazolo[1,5-c]naphth[2,1-e]-1,3-oxazine}-2,5’-dione* (**17**): Obtained from **1** and one equivalent of triphosgene using the procedure described for **9**. Yield 69%, reddish solid, mp 207 °C; IR: 3,256 (NH), 1,748, 1,755 (2C=O); ^1^H-NMR: 2.12 (s, 3H), 3.58 (d, *J* = 14.70 Hz, 1H), 3.65 (d, *J* = 14.70 Hz, 1H), 6.72 (m, 4H), 6.97 (m, 4H), 7.11 (m, 5H), 7.58 (m, 5H), 7.91 (m, 2H), 8.40 (m, 2H); ^13^C-NMR: 32.15, 59.65, 66.06, 85.55, 112.48, 112.93, 113.91, 116.47, 116.80, 119.92, 120.36, 121.68, 122.19, 122.23, 124.96, 125.49, 127.55, 127.68, 128.81, 129.19, 133.56, 133.86, 134.09, 141.92, 142.46, 142.78, 143.18, 145.29, 145.42, 145.69, 149.24, 149.90; MS: m/z (%) 471 (M^+^ - C_6_H_5_N_2_CO, 20), 455 (M^+^ - C_6_H_5_N_2_CO_2_, 14), 440 (57), 423 (28), 351 (12), 302 (21), 259 (100), 258 (17), 230 (34), 185 (29), 168 (39), 128 (21), 115 (38), 93 (70), 77 (51), 63 (35); Anal. Calcd. for C_38_H_28_N_4_O_4_: C, 75.48; H, 4.66; N, 9.26. Found: C, 75.21; H, 4.71; N, 9.19.

*3’-(4-Chlorophenyl)-3-[(4-chlorophenyl)amino]-1’,12’b-dihydro-12’-b-methylspiro{4H-naphth[1,2-e]-1,3-oxazine-4,2’(3’H)-[5H]pyrazolo[1,5-c]naphth[2,1-e]-1,3-oxazine}-2,5’-dione* (**18**): This compound was obtained from **2** and one equivalent of triphosgene using the procedure described for **9**. Yield, 72%, mp 267°C; IR: 3,219 (NH), 1,724, 1,753 (2C=O); ^1^H-NMR: 2.10 (s, 3H), 3.49 (d, *J* = 14.70 Hz, 1H), 3.60 (d, *J* = 14.70 Hz, 1H), 6.35 (m, 3H), 6.65 (m, 4H), 6.93 (m, 4H), 7.66 (m, 5H), 7.82 (m, 2H), 8.40 (m, 2H); ^13^C-NMR: 32.08, 60.07, 66.05, 85.47, 111.88, 114.22, 117.91, 119.62, 121.02, 121.54, 122.16, 122.56, 123.11, 125.25, 125.57, 126.45, 127.18, 127.40, 127.47, 127.66, 127.78, 127.93, 128.81, 128.84, 128.91, 133.47, 134.31, 141.39, 141.81, 143.90, 145.61, 149.61, 149.01, 149.07; MS: m/z (%) 505 (M^+^- ClC_6_H_4_N_2_CO, 22), 489 (M^+^- ClC_6_H_4_N_2_CO_2_, 100), 410 (7), 336 (13), 295 (12), 259 (21), 230 (10), 168 (26), 139 (24), 127 (28), 115 (21), 99 (9), 90 (5), 76 (15), 63 (12); Anal. Calcd. for C_38_H_26_Cl_2_N_4_O_4_: C, 67.76; H, 3.89; N, 8.32. Found: C, 67.46; H, 3.68; N, 8.54.

*3’-(4-Bromophenyl)-3-[(4-bromophenyl)amino]-1’,12’b-dihydro-12’b-methylspiro{4H-naphth[2,1-e]-1,3-oxazine-4,2’(3’H)-[5H]pyrazolo[1,5-e]naphth[2,1-e]-1,3-oxazine}-2,5’-dione* (**19**): Obtained from **3** and one equivalent of triphosgene using the procedure described for **9**. Yield, 71%, mp 246 °C; IR: 3,232 (NH), 1,730, 1,745 (2C=O); ^1^H-NMR: 2.09 (s, 3H), 3.44 (d, *J* = 14.70 Hz, 1H), 3.54 (d, *J* = 14.70 Hz, 1H), 6.28 (m, 3H), 6.60 (m, 3H), 6.85 (m, 2H), 7.04 (m, 2H), 7.52 (m, 6H), 7.78 (m, 2H), 8.34 (m, 2H); ^13^C-NMR: 32.04, 60.11, 65.96, 85.41, 111.75, 113.97, 114.67, 115.87, 117.98, 118.21, 119.56, 121.00, 121.53, 122.16, 122.55, 123.11, 125.31, 127.42, 127.48, 127.69, 127.79, 127.95, 128.94, 131.70, 131.74, 132.05, 133.66, 134.31, 141.78, 141.88, 144.40, 148.95, 149.15; MS: m/z (%) 549 (M^+^- BrC_6_H_4_N_2_CO, 6), 533 (M^+^- BrC_6_H_4_N_2_CO_2_, 100), 409 (15), 382 (20), 380 (17), 340 (24), 339 (85), 258 (86), 202 (24), 196 (51), 168 (74), 129 (59), 115 (84), 99 (51), 91 (49), 90 (52), 76 (71), 63 (84); Anal. Calcd. for C_38_H_26_Br_2_N_4_O_4_: C, 59.86; H, 3.43; N, 7.34. Found: C, 59.73; H, 3.49; N, 7.42.

*3’-(4-Chlorophenyl)-3-[(4-chlorophenyl)amino]-1’,12’b-dihydro-12’b-methylspiro{4H-naphth[1,2-e]-1,3-oxazine-4,2’(3’H)-[5H]pyrazolo[1,54-c]naphth[1,2-e]-1,3-oxazine}-2,5’-dione* (**20**): Obtained from **6** and one equivalent of triphosgene using the procedure described for **9**. Yield, 55%; mp 227 °C; IR: 3,338 (NH), 1,730, 1,755 (2C=O); ^1^H-NMR: 2.51 (s, 3H), 3.41 (d, *J* = 14.65 Hz, 1H), 3.54 (d, *J* = 14.65 Hz, 1H), 7.07 (m, 3H), 7.25 (m, 4H), 7.51 (m, 5H), 8.11 (m, 4H), 8.29 (m, 2H), 8.61 (m, 2H); ^13^C-NMR: 17.20, 27.87, 88.06, 97.68, 114.00, 114.31, 114.46, 115.89, 116.26, 120.55, 123.56, 124.20, 124.87, 125.67, 125.73, 127.89, 128.57, 128.72, 128.87, 128.93, 129.76, 131.21, 131.32, 131.66, 131.77, 131.80, 136.60, 138.88, 143.81, 144.17, 145.50, 147.04, 148.60, 149.01; MS: m/z (%) 505 (M^+^- ClC_6_H_4_N_2_CO, 20), 489 (M^+^- ClC_6_H_4_N_2_CO_2_, 100), 410 (7), 336 (21), 319 (15), 295 (21), 293 (44), 258 (31), 230 (14), 139 (29), 127 (44), 115 (35), 75 (20), 63 (16); Anal. Calcd. for C_38_H_26_Cl_2_N_4_O_4_: C, 67.76; H, 3.89; N, 8.32. Found: C, 67.53; H, 3.93; N, 8.46.
